# Kaposiform hemangioendothelioma presented with raynaud phenomenon: a case report

**DOI:** 10.1186/s12887-023-04407-1

**Published:** 2023-11-17

**Authors:** Lingke Liu, Weizhong Gu, Liping Teng, Yiping Xu, Fei Zheng, Minfei Hu, Meiping Lu, Xuefeng Xu

**Affiliations:** 1 Department of Rheumatology Immunology & Allergy, The Children’s Hospital, Zhejiang University School of Medicine, National Clinical Research Center for Child Health, Binsheng Rd 3333, Binjiang District, Hangzhou, 310052 P.R. China; 2 Pathology, National Clinical Research Center for Child Health, Hangzhou, 310003 PR China; 3 Pediatrics, The Affiliated Hospital of Shaoxing University, Shaoxing, 312000 PR China

**Keywords:** Case report, Children, Kaposiform hemangioendothelioma, Raynaud phenomenon, Thrombocytopenia

## Abstract

**Background:**

Kaposiform hemangioendothelioma (KHE) is a rare vascular neoplasm affecting infants or young children. KHE includes a spectrum of lesions, ranging from small and superficial tumors to large and invasive lesions with Kasabach-Merritt phenomenon (KMP). Currently, no published studies have reported a KHE presenting as thrombocytopenia and Raynaud phenomenon.

**Case presentation:**

A 2-year-old boy with right hand swelling and thrombocytopenia was admitted to our hospital. His right hand turned swelling and red, even occasionally cyanotic. This condition became worse in response to cool environments and improved with warming, and platelet counts were between 50 ~ 80 × 10^9/L. Physical examination on admission revealed the swelling and frostbite-like rash of the right-hand fingers, and the skin temperature of the right hand was lower than the left. On day 3 of admission, chest CT results showed an irregular mass on the right side of the spine. The puncture biopsy demonstrated positive CD31, D2-40, and FLI1 immunohistochemical staining, but negative GLUT1 staining, confirming the diagnosis of KHE. Furthermore, endothelin-1 (ET1) expression levels significantly increased, and eNOS and A20 expression levels significantly decreased comparing with control patients. The patient received methylprednisolone and sirolimus treatments, and his condition gradually improved during the follow-up.

**Conclusions:**

We reported the first case of KHE presenting with thrombocytopenia and Raynaud phenomenon. The development of Raynaud phenomenon could be associated with increased ET-1 and reduced eNOS and A20 expressions. Careful differential diagnosis of hidden KHE should be considered in children with thrombocytopenia and Raynaud phenomenon.

## Introduction

Kaposiform hemangioendothelioma (KHE) is a rare vascular neoplasm affecting infants or young children, even the embryonic period with locally aggressive characteristics [1–3]. KHE is usually manifested as an infiltrative growing soft tissue mass, located on the skin surface or deeper in the extremities, torso (including intrathoracic cavity and retroperitoneum), and the cervicofacial region [4–6]. Currently, there is little literature specifically on the incidence rate of KHE. Croteau et al. reported that the annual prevalence and incidence rates in Massachusetts were estimated to be 0.91 and 0.071 cases per 100,000 children, respectively [6]. Complications of KHE are common, including the Kasabach-Merritt phenomenon (KMP), musculoskeletal disorders, lymphedema, and compression of vital structures [5]. KMP refers to the clinical symptom spectrum of thrombocytopenia, consumptive coagulopathy and purpura related to KHE or tufted angioma, complicating infantile hemangioma [7]. KMP is present in more than 90% of patients with retroperitoneal tumors and in all patients with extra-regional tumors [4]. In an 18-month follow-up of 191 patients, about 80% survived with no evidence of disease or with residual non-active tumor. Unfortunately, the prognosis was worse for patients with KHE located in the retroperitoneum, and KHE complicated by KMP had a higher mortality rate compared with those without KMP [4].

KHE may rarely present also as an excessive vascular mass leading to distal intestinal obstruction [8]. Musculoskeletal complications including decreased range of motion and bone-joint changes can develop early in the disease course of KHE [9]. In the current literature, there is no report regarding the definite paraspinal KHE manifesting as both frostbite-like rash and Raynaud phenomenon of hands. Here, we reported a rare pediatric case with KHE, presenting with Raynaud phenomenon and swelling and frostbite-like rash of the right hand. This present report aims to make pediatricians pay more attention to the complexity of pediatric KHE.

## Case presentation

**Fig. 1:**
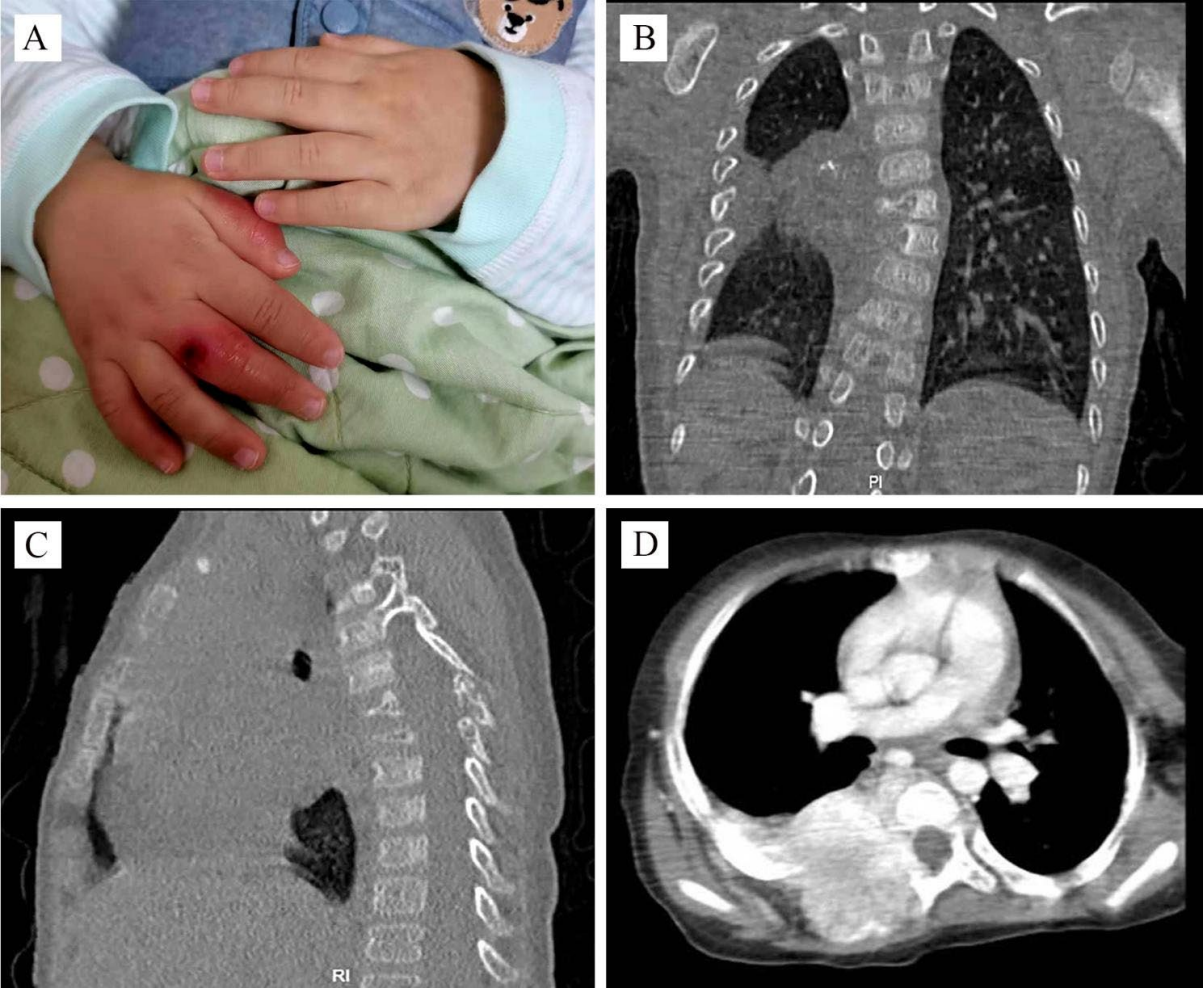
**(A)** Noted the swelling and frostbite-like rash of the right hand. The chest CT (**B** and **C**) showed an irregular mass on the right side of the spine (T3-6 vertebral level), presenting with bone destruction of vertebral, attachments, and multiple ribs. The enhanced CT **(D)** scan shows an enhanced soft-tissue mass extending to the posterior thoracic musculature and the spinal canal

In February 2022, a 2-year-old boy with right-hand swelling and thrombocytopenia was admitted to the Department of Rheumatology Immunology & Allergy. One year ago, his parents found that his right hand turned swelling and red, even occasionally cyanotic. This condition became worse in response to cool environments and improved with warming. Additionally, blood routine examination indicated that platelet counts were between 50 ~ 80 × 10^9/L. The boy did not accept other therapies except for the avoidance of cold temperatures. Physical exam on admission showed visible tonsils; unrecognized rales in both lungs; no murmurs in his heart; abdominal softness, no hepatosplenomegaly; no positive signs on the nervous system; muscle strength examinations had no abnormal findings. The swelling and frostbite-like rash of the right-hand fingers were observed (Fig. 1A). Furthermore, the skin temperature of the right hand was significantly lower than the left. The radial pulse had identical shape on both sides.

**Fig. 2:**
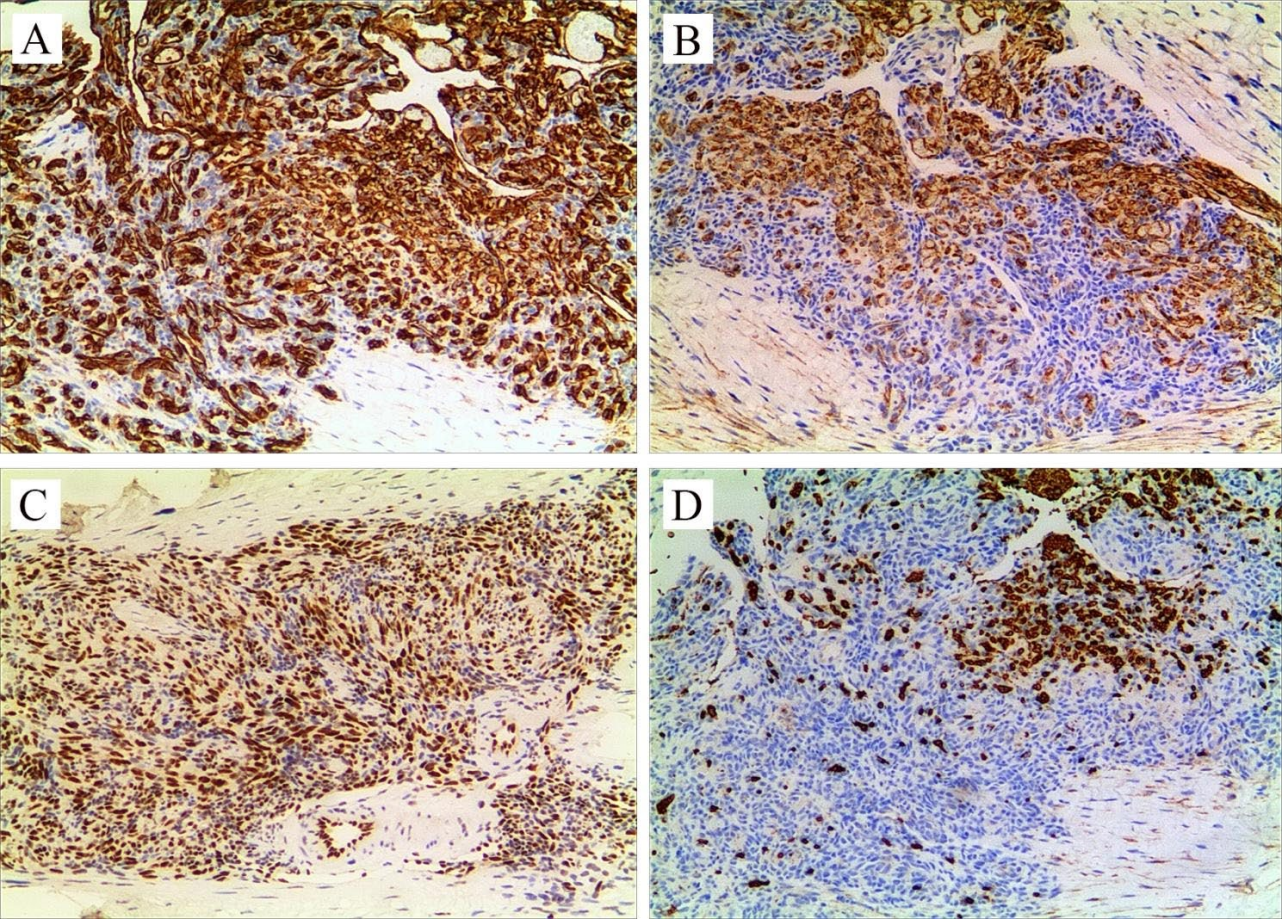
Immunohistochemical staining shows an abnormal proliferation of spindle cells and well-formed capillary-like vessels. The significant positive CD31 **(A)**, D2-40 **(B)**, and FLI1 **(C)** immunohistochemical staining, but negative GLUT1 (D) staining were observed. Erythrocytes with positive Glut1 **(D)** expressions were seen in the channels

Laboratory findings are shown in Table 1. Peripheral blood count showing WBC 11.95 × 10^9/L, neutrophils 44.2%, Hgb 132 g/L, platelet count 54 × 10^9/L. The coagulation function revealed fibrinogen 1.55 g/L and D-dimer 17.32 mg/L. Liver function, renal function, thyroid function, immunoglobulins, and electrolytes were normal. Hepatitis B and C viruses and human immunodeficiency viruses were all negative. Bone marrow aspiration revealed that the number of megakaryocytes increased, and the platelet production function was normal without leukemic changes and hemophagocytosis. On day 3 of admission, we noted that the boy had a lower right shoulder while walking. We suspected that the child could have scoliosis, and performed a chest CT. The chest CT results showed an irregular mass on the right side of the spine (T3-6 vertebral level) with about 31.3*31.7 mm (Fig. 1B-D). The enhanced CT demonstrated that T3-6 vertebrae and attachments, and multiple ribs had bone destruction, some mass appeared to extend to the spinal canal, even the right spine. The chest CT demonstrated no hemorrhagic or cystic zones. The boy was suspected of KHE and transferred to the surgical oncology department, performing an ultrasound-guided puncture biopsy. Pathological results demonstrated positive CD31, D2-40 (Podoplanin), and FLI1 (Friend leukemia integration 1) immunohistochemical staining on biopsy specimen, but negative GLUT1 (Glucose transporter protein isoform 1) staining, further confirming the diagnosis of KHE (Fig. 2). Considering the high risk of surgery, the patient’s parents received methylprednisolone (4 mg/kg/day) and sirolimus (0.8 mg/m^2^/dose twice daily) treatments. The patient’s platelets remained stable within the normal range and his right hand gradually improved during the follow-up. After one year of treatment, the patient’s right hand swelling significantly improved, and chest X -ray revealed that tumor became smaller. Additionally, scoliosis is currently being corrected.

**Table 1:** Blood laboratory findings on admission

Variables	Values	Reference value
White blood cell (10^9/L)	11.95	4–12
Neutrophils (10^9/L)	5.29	1.5–7.8
Hemoglobin (g/L)	132	110–155
Platelet (10^9/L)	54	100–400
Reticulocyte %	1.5	0.5–1.5
C reactive protein (mg/L)	0.25	0.00–8.00
Total protein (g/L)	71.7	57–80
Albumin (g/L)	46.3	32–52
Globulin (g/L)	25.4	20–40
Alanine aminotransferase (U/L)	16	< 50
Aspartate aminotransferase (U/L)	43	15–60
Lactate dehydrogenase (U/L)	354	110–295
Alkaline phosphatase (U/L)	262	42–362
Creatine kinase (U/L)	269	39–308
Creatinine (µmol/L)	29	11–34
Urea (mmol/L)	6.7	2.8–7.6
CD19%	25.6	18.5–28.0
CD3%	61.5	56–68
CD4%	32.7	29–40
CD8%	22.6	19–25
CD3-CD16 + CD56+	14.6	9–19
IL 2 (pg/ml)	2	1.1–9.8
IL 4 (pg/ml)	4.5	0.1-3.0
IL 6 (pg/ml)	5.4	1.7–16.6
IL 10 (pg/ml)	6.0	2.6–4.9
Tumor necrosis factor (pg/ml)	2.8	0.1–5.2
Interferon-γ (pg/ml)	4.2	1.6–17.3
IgG (g/L)	8.4	3.82–10.58
IgA (g/L)	0.73	0.14–1.14
IgM (g/L)	0.90	0.4–1.28
IgE (IU/ml)	53.1	0.0-100.0
Complement 3 (g/L)	1.28	0.90–1.80
Complement 4 (g/L)	0.38	0.10–0.40
Antinuclear antibody	1:80	< 1:80
Anti-dsDNA (IU/ml)	< 100	< 100
PAC-1 + CD61+/CD19+ (%)	0.00	
CD19 + CD61+/CD19+ (%)	1.00	
CD61+/CD42+ (%)	98.0	> 30
CD41a+/CD42+ (%)	97.7	> 30
Fibrinogen (g/L)	1.55	1.8-4.0
D-dimer (mg/L)	17.32	< 0.55

**Fig. 3:**
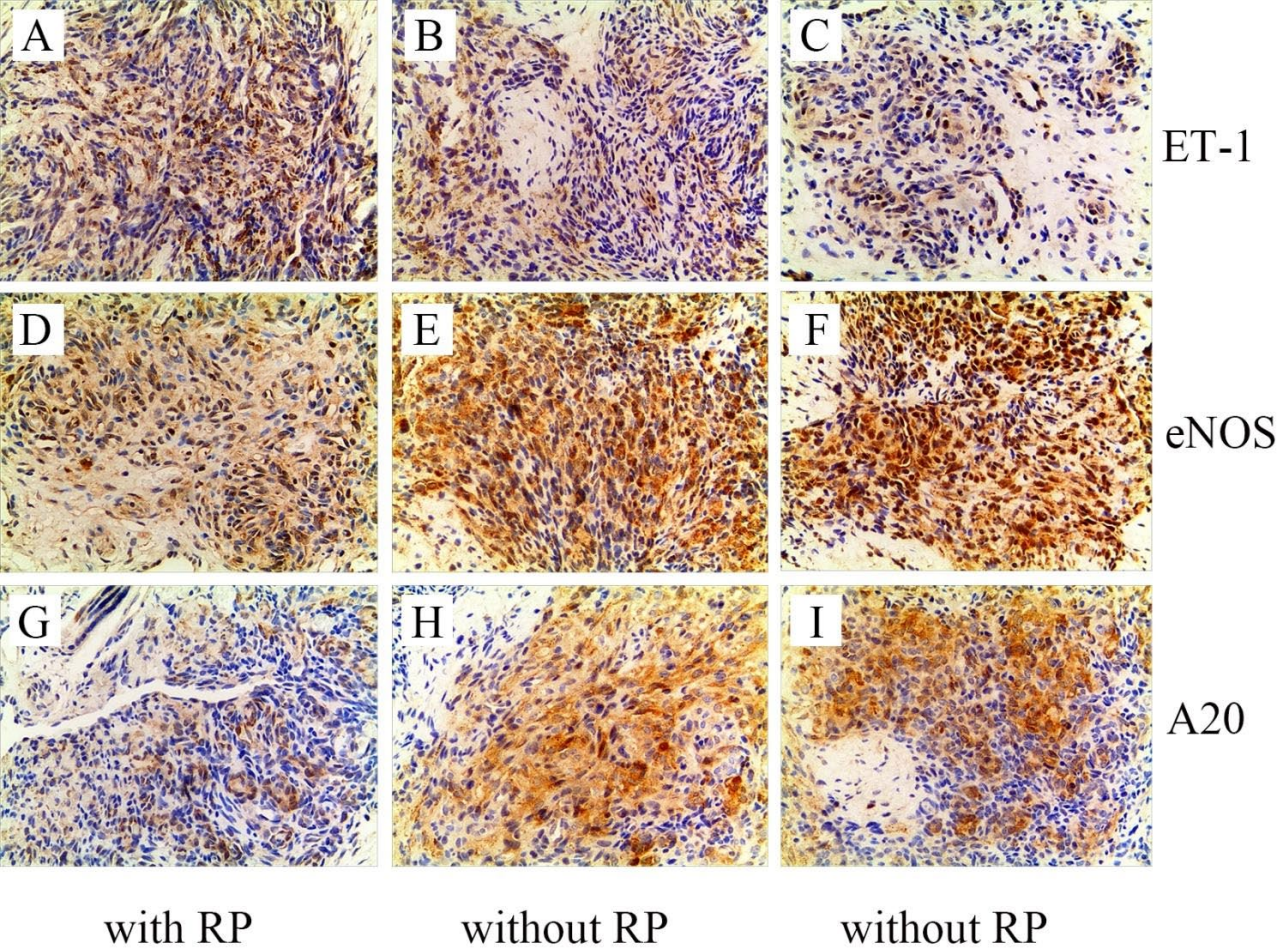
Representative staining images of ET-1, eNOS and A20 in the three cases. The significant ET1 expression was observed in our case **(A)** relative to other two KHE patients without Raynaud phenomenon (**B** and **C**). The expressions of eNOS **(D)** and A20 **(G)** were reduced in our case relative to other two KHE patients without Raynaud phenomenon (eNOS for **E** and **F;** A20 for **H** and **I**). eNOS = endothelial NO synthase; ET-1 = endothelin 1; RP = Raynaud phenomenon

In addition to reduced platelet counts, the boy had a special Raynaud phenomenon. Therefore, we speculated that this patient could have vasomotor dysfunction. Because endothelin-1 (ET1) and endothelial nitric oxide synthase (eNOS) play an important role in the contraction and relaxation of blood vessels, respectively [10]. Moreover, ubiquitin-modifying protein A20 overexpression in endothelial cells can significantly increase basal eNOS mRNA and protein levels [11]. Thus, immunohistochemical staining for ET1, eNOS, and A20 was performed on the biopsy specimen. Two previous cases without Raynaud phenomenon were chosen as a control group. Interestingly, we found that ET1 expression levels significantly increased, and eNOS and A20 expression levels significantly decreased compared with control patients (Fig. 3).

## Discussion

KHE was first designated by Zukerberg and coworkers in 1993 as an entity distinct from infantile hemangioma because of its locally invasive growth, aggressive course and focal Kaposi-like appearance [12]. Since then, KHE with different clinical manifestations and large cohort studies have been reported [4, 6, 8, 13–15]. KHE can occur in various locations and present as highly infiltrative and heterogeneous masses invading the skin, adjacent muscles, and bones [16]. Currently, vascular tumors are divided into benign, locally aggressive, and malignant entities. KHE is classified as locally aggressive or borderline vascular tumors [17]. Clinically, KHE has high mortality rates, mainly due to local invasive features, compressive effects, or the life-threatening consumable coagulation disease known as KMP [5, 7].

The clinical manifestations of KHE vary from small superficial tumors without KMP to large invasive lesions with KMP. High-resolution CT or MRI contribute to detecting the concealed KHE mass in deep tissues [16]. If the KHE is highly suspected and the CT results are normal, MRI examination may also be considered to confirm the presence of KHE [18]. Qiu et al. demonstrated the advantage of 18 F-FDG PET/CT in evaluating the disease activity of KHE and guiding therapy and prognostication [19]. More than two-thirds of KHE developed KMP and 10% lacked cutaneous findings. Furthermore, retroperitoneal and intrathoracic KHE are most likely to develop KMP [6]. Patients with KMP were much more likely to have damages involving trunk compared with those without KMP [20]. KHE without cutaneous involvement could be associated with severe complications [20]. Our case presented with the Raynaud phenomenon and swelling of the right hand and thrombocytopenia in the early stage of the disease. KHE failed to be early diagnosed due to a lack of local mass or skin lesion in the right hand or upper limb. During this hospitalization, the chest CT showed an irregular mass on the right side of the spine, and subsequently puncture biopsy specimen confirmed the diagnosis of KHE. Our case had thrombocytopenia, hypofibrinogenemia, and increased dimers, indicating the presence of KMP. These symptoms significant improved after glucocorticoid therapy.

Fewer cases of KHE and vertebral involvement and scoliosis have been reported. Shyam et al. reported a case manifesting with painless scoliosis with no restriction of movement or neurological abnormality [21]. Chin and his coworkers described a KHE case presenting with progressive fixed hyperlordotic deformity, multiple non-specific spinal lesions, and abnormal blood tests [22]. Other authors also reported KHE cases with progressive painless scoliosis, even misinterpreted as adolescent idiopathic scoliosis [23, 24]. Our case presented with vertebral involvement, associated with scoliosis and absence of characteristic skin lesions, further enriching scoliosis etiology.

The feature of the pathology of KHE is lobular, infiltrative endothelial cell bundles, hyperemic capillaries, slit-like vascular spaces, and epithelioid endothelial cells [25]. The spindle endothelial cell component in KHE is mainly solid and confluent vascularized nodules with microthrombus and fibrins [26], and typically evoking a dense hyaline stromal response [14]. In addition to a characteristic spindle-shaped endothelial cell nodules forming slit-like vascular channels, KHE also has positive lymphatic marker D2-40 [26]. Moreover, endothelial cells in nodules have positive CD31, CD34, and FLI1 expressions but negative GLUT1 expression [14], while erythrocytes in the slit-like channels can express GLUT1 [27]. For the present case, immunohistochemical staining showed positive CD31, D2-40, and FLI1 expressions and negative GLUT1 expression in the spindle cells, further confirming the diagnosis of KHE.

Raynaud phenomenon is an excessive physiological response of the limbs to cold or emotional stress. Many conditions including systemic sclerosis, systemic lupus erythematosus, vasculitis, dermatomyositis, and mixed connective tissue disease can result in the Raynaud phenomenon [28]. The pathogenesis of the Raynaud phenomenon is very complex, which is related to abnormal vascular wall and neural regulation, and abnormal interaction of intravascular factors [29]. Raynaud phenomenon developed when the weak balance between vasodilatation and vasoconstriction is destroyed in favor of vasoconstriction [29]. Dysfunctional endothelial cells have decreased activity of vasodilators (nitric oxide) and can also overexpress thrombotic and inflammatory activity, including the release of the vasoconstrictor ET1 [28]. Based on the pathogenesis of Raynaud phenomenon, it is reasonable to speculate that the Raynaud phenomenon in this boy could be strongly associated with the imbalance between vasodilators and vasoconstrictors. Interestingly, our immunohistochemical staining results demonstrated increased ET1 and decreased eNOS expressions in the biopsy specimen, indicating the predominant role of vasoconstrictors instead of dilatators. In addition, reduced ubiquitin-modifying protein A20 expression in endothelial cells may contribute to decreasing eNOS expression, indirectly enhancing the vasoconstrictor effect. Taken together, increased ET-1 and reduced eNOS and A20 expressions may be closely associated with the development of KHE.

The best therapeutic method is to completely remove the mass, because it is effective for most patients [4]. There is a lack of well-designed clinical trials and insufficient evidence to provide drug recommendations for KHE [5]. In 2013, consensus treatment statements by *Drolet et al.*. recommended the combination therapy of corticosteroids and vincristine for the management of KHE [15]. However, vincristine requires central venous access, which probably limits its use. The deletion of mTOR (mammalian target of rapamycin)-related proteins tuberous sclerosis complex 2 and phosphatase and tensin homologue would lead to abnormal activation of the mTOR signaling pathway and may involve the pathogenesis of KHE, suggesting sirolimus as a good therapeutic choice [30]. Currently, sirolimus plus steroids is considered as a first-line therapy for the treatment of KHE with or without KMP compared with vincristine plus steroids [5]. Given the high risk of surgery, our case received methylprednisolone and sirolimus treatments. The patient’s platelets remained stable, and his right hand gradually improved during the follow-up.

## In conclusion

We reported the first case of KHE manifesting as thrombocytopenia and the Raynaud phenomenon. The development of the Raynaud phenomenon in this boy could be associated with increased ET-1 and reduced eNOS and A20 expressions. The diversity of clinical manifestations could make the diagnoses of pediatric KHE more complicated. Careful differential diagnosis of hidden KHE should be considered in children with thrombocytopenia and the Raynaud phenomenon.

## Data Availability

The original contributions presented in the study are included in the article, further inquiries can be directed to the corresponding author.

## Abbreviations

A20: ubiquitin-modifying protein
CD: Cluster of Differentiation
eNOS: endothelial nitric oxide synthase
ET1: endothelin 1
Ig: immunoglobulin
IL: interleukin
KHE: Kaposiform hemangioendothelioma
KMP: Kasabach-Merritt phenomenon
WBC: white blood cell
